# Identifying metrics of success for transitional care practices in childhood cancer survivorship: a qualitative interview study of survivors

**DOI:** 10.1186/s12885-020-07360-9

**Published:** 2020-09-21

**Authors:** Karim Thomas Sadak, Milki T. Gemeda, Michelle Grafelman, Joseph P. Neglia, David R. Freyer, Eileen Harwood, Jude Mikal

**Affiliations:** 1 University of Minnesota Masonic Children’s Hospital, University of Minnesota Masonic Cancer Center, 420 Delaware St. SE - Mayo MMC 484, Minneapolis, MN 55455 USA; 2 University of Minnesota Medical School, 420 Delaware St. SE - Mayo MMC 484, Minneapolis, MN 55455 USA; 3 University of Minnesota, 420 Delaware St. SE - Mayo MMC 484, Minneapolis, MN 55455 USA; 4 Children’s Center for Cancer and Blood Diseases, Children’s Hospital Los Angeles, USC Norris Comprehensive Cancer Center; California, 4650 Sunset Blvd. MS #54, Los Angeles, CA 90027 USA; 5 Departments of Pediatrics and Medicine, Keck School of Medicine, University of Southern, California 4650 Sunset Blvd. MS #54, Los Angeles, CA 90027 USA; 6 Division of Epidemiology & Community Health, University of Minnesota School of Public Health, 420 Delaware St. SE - Mayo MMC 484, Minneapolis, MN 55455 USA; 7 Division of Health Policy and Management, University of Minnesota School of Public Health, 420 Delware St. SE, MMC729, Minneapolis, MN 55455 USA

**Keywords:** Childhood cancer survivor transition qualitative

## Abstract

**Background:**

Adolescent and young adult (AYA) childhood cancer survivors (CCS) should be empowered to continue their survivor-focused care as they transition into adult medicine. However, the majority of AYA-aged survivors become lost to follow up around the age of typical transition to adulthood. The purpose of this study was to identify, from the patient’s perspective, key factors that facilitate successful transitions to adult-centered survivorship care.

**Methods:**

A qualitative study was conducted with AYA CCS (*n* = 29) from the survivorship clinic of a single institution as key informants. Data were collected through a series of structured phone interviews and subjected to thematic content analysis.

**Results:**

Four major themes with multiple subthemes were identified: (1) transition practices need to be flexible and individually tailored; (2) effective communication is critical to a successful transition; (3) continuity in providers is needed during the transition; and (4) comprehensive care means care that also addresses psycho-social well-being.

**Conclusions:**

From the perspective of AYA CCS, the ideal model of transitional survivorship care could include a patient navigator who promotes provider flexibility, consistent communication, and pro-active comprehensive care that encompasses both medical and psycho-social well-being. Models of care for CCS should be built to provide, or seamlessly facilitate, continuous survivor-focused care across the age continuum. A longitudinal relationship with a survivor-focused provider can help promote the values that CCS’ report as important in transitioning care from pediatric- to adult-centered care.

## Background

There are currently more than 419,000 childhood cancer survivors (CCS) alive in the US, most of whom are adolescent and young adult (AYA) age [1, 2]. While the Institute of Medicine recommends life-long surveillance and screening interventions for most, a landmark study by *Nathan* et al. [3] found that only 18% of these AYA cancer survivors report receiving adequate follow-up care related to their childhood cancer. As adolescents gain personal independence and assume greater responsibility for their own healthcare, there is a significant risk for critical follow up services being lost. This interruption of survivor-focused care prevents appropriate surveillance for late effects of cancer treatment, which may develop any time after treatment including the AYA years and into middle adulthood [4–11]. As a result, it is imperative to support AYA survivors of pediatric cancer to continue their survivor-focused care as they transition into adult medicine to promote the life-long continuation of survivor-focused care.

Given that the majority of these AYA patients are lost to follow up around the age of typical transition to adulthood, it is critical to construct models of care to guide follow up care throughout the transition from adolescence into adulthood. However, currently no evidence-based model exists. Care for AYA CCS are widely disparate across the world [12–15] and providers lack the knowledge, skills, and institutional resources to monitor patients through this critical transition [16–18]. The Society for Adolescent Medicine and several professional societies in pediatrics, internal medicine and family medicine have operationalized the key principles for successful transitions to adult care [19–21]. Despite all of this, most CCS do not effectively transition to adult-centered care for the necessary screening tests and late effects counselling [3, 12, 16, 22]. The reasons for this are unclear and understudied.

To create an optimal model for the transition of survivor-focused care, additional findings are needed to inform the delivery of care. Findings must reflect the care preferences from all stakeholders: survivors, parents, members of the healthcare team (both pediatric and adult), and healthcare administrators. There is data from select stakeholders to help inform the creation of transitional care models for CCS, including a previous study that reported the perspective of survivor-focused providers [23]. This was the first in a series of studies identifying the best practices for delivering care to AYA-aged CCS. Our current study is the continuation of this effort sought to determine what best practices look like from the vantage point of the AYA-aged CCS. The purpose of this study was to identify the factors that ensured or that patients felt would be necessary to ensure successful transitions through optimal models of care to adult-centered survivor-focused follow up as reported by AYA-aged CCS.

## Methods

### Study design

A qualitative study was conducted with childhood cancer survivors as key informants. More details on study design can be found in *Sadak* et al [23]. In order to understand indicators of success in transitional care practices as reported by AYA-aged CCS, we conducted a series of semi-structured qualitative interviews. This had previously been done with providers caring for CCS and is currently taking place with a cohort of parents of CCS [23]. For this study, a structured interview protocol was selected to promote reliability as the interview questions were administered to various populations (e.g. providers, survivors and parents).

### Participant recruitment

In accordance with Institutional Review Board (IRB) guidelines, participants were recruited during routine outpatient long-term follow up (LTFU) care appointments. Participants came from a convenience sample of patients receiving survivor-focused care at our institution. Survivors had a mix of health insurance plans including both private and public payers which was reflective of the general population served by this clinical program and consistent with general U.S. insurance practices. Personal health information was not collected to ensure anonymity and promote candid responses to all interview questions and all data was aggregated for analysis as a group. Patients were provided study details including information on participation incentives ($10 retail gift card) and were provided contact information for study personnel. Interested patients were invited to contact the study team directly via phone or email to ask any follow-up questions and to schedule what was expected to be a 15–30 min phone interview at their convenience.

To be eligible for inclusion in the study, participants were required to be childhood cancer survivors currently between the ages of 18 and 29, and to be receiving outpatient care in the Childhood Cancer Survivor Program (CCSP) at the University of Minnesota (either in the pediatric or adult setting). Survivors were considered ineligible only if they did not speak English or if they had a health condition that prevented them from completing a phone interview. Participant recruitment took place between April and July 2016. Our goal was to interview 25 participants, per standard phenomenological requirements to achieve informational redundancy and theoretical saturation of the desired content [24]. A total of 30 eligible survivors were approached and we obtained a final sample size of 29, of whom 18 were still in pediatric care settings, while 11 had already transferred to adult care settings.

### Data collection

We began each interview with a review of their rights as research participants to establish trustworthiness, transparency and credibility. To ensure that participants understood key terms and concepts, we then reviewed key terms, including “transition.” We defined “transition” as the gradual change from a pediatric, or child-centered, setting to an adult-focused health care setting in order to continue cancer-related follow-up care. Participants were informed that they could drop out of the study at any point, including mid-interview, and still receive their participant incentive in an effort to build credibility and trustworthiness. It was explained to all participants that the interviews would be aggregated and anonymous to help provide transparency with our study methods and analysis plans.

The interview protocol (Online Resource 1) was designed to elicit spontaneous narrative that considered the perspective of survivors still receiving pediatric-focused care and also those that had been experienced transition success to adult-centered care. Narrative and short-answer data were collected using a semi-structured interview protocol that consisted of one grand-tour question: “Thinking about your experience receiving care in the CCSP, how would you describe what you consider to be a successful transition?” Trained interview staff were also encouraged to use mini-tour or probing questions to follow up on rich points and conceptualizations of key terms. They had no prior knowledge of childhood cancer survivorship care to minimize potential biases but were provided a general orientation to the language and terminology likely to be used by the participants.

While interviews varied in duration from 10 to 45 min, each participant was encouraged to speak on system or support features that promote successful transitions in care settings from the perspectives of various stakeholders. Probing questions were revised by author KS between interviews to ensure that a comprehensive understanding of the issues were elicited. The interviewer and author KS met after every 5 interviews to discuss any patterns or highlighted areas of inquiry. Interviews were audio-recorded using standard phone interview recording devices and transcribed using Tybee Types, a professional transcription service. No repeat interviews were needed. Transcriptions generated a total of 191 pages of text that were then redacted to remove any information that could be used to identify participants. Redacted transcripts were then uploaded to NVivo 9.0.

### Analysis

Principles of directed content analysis were used to identify major themes in the data [23, 25]. Initial codes were identified through an inductive approach where authors KS, JM, and MG each reviewed 2 transcripts to decide on a coding scheme. Research bias was also considered at the onset of this study, particularly with the selection of coders and methodologies for qualitative data analysis. The study team conducting this research did not want to influence the results in order to portray a certain outcome. The first and second author read and coded each key informant transcript and met periodically with the study team to discuss expected and evolving themes, codes, interpretations, and quotes. Multiple coders were used (KTS and MG) throughout the process to also minimize confirmation bias and furthermore, all prevailing themes were reevaluated by other members of the study team who had expertise in qualitative research methodologies (JM) but no experience delivering clinical care (MTG) to help deal with any potential research bias. This resulted in an iterative review of the identified themes that was central to ensuring that rigor was maintained throughout the process. NVivo 9.0 software was used to organize transcribed data for the study team to complete the analyses.

## Results

### Participants

The study sample included 29 key informants, all from the Upper Midwest region of the United States. All were patients currently receiving survivor-focused care and additional descriptive information on the study sample is reported in Table 1. Age and sex of participants has been removed to help maintain anonymity and age range has been used instead.

**Table 1:** Descriptive information of interview participants

Participant	Age Range (years)	Pre/Post Transition	Diagnosis
1	25–27	Post	Leukemia
2	25–27	Post	Lymphoma
3	25–27	Post	Leukemia
4	25–27	Post	Lymphoma
5	25–27	Pre	Sarcoma
6	18–19	Pre	Lymphoma
7	> 27	Post	Sarcoma
8	22–24	Pre	Sarcoma
9	25–27	Post	Lymphoma
10	20–21	Pre	Sarcoma
11	22–24	Pre	Hematologic Condition Treated with Bone Marrow Transplant
12	25–27	Pre	Sarcoma
13	22–24	Pre	Sarcoma
14	> 27	Post	Leukemia
15	20–21	Pre	Central Nervous System Malignancy
16	22–24	Post	Central Nervous System Malignancy
17	20–21	Pre	Leukemia
18	25–27	Pre	Leukemia
19	22–24	Pre	Leukemia
20	18–19	Pre	Leukemia
21	20–21	Pre	Neuroblastoma
22	20–21	Pre	Central Nervous System Malignancy
23	18–19	Pre	Hematologic Condition Treated with Bone Marrow Transplant
24	20–21	Post	Lymphoma
25	22–24	Pre	Hematologic Condition Treated with Bone Marrow Transplant
26	25–27	Post	Sarcoma
27	> 27	Post	Lymphoma
28	25–27	Pre	Lymphoma
29	22–24	Pre	Sarcoma

### Themes

Survivors were asked to describe desired practices for the transition of care from pediatric- to adult-centered care settings when completing the phone interview. Data analysis resulted in 4 major themes as described in Table 2.

**Table 2:** Summary of Themes and Subthemes with Sample Quotes

Theme / Subtheme	Sample Quotes
1. Transition practices should be flexible and individually tailored	
1a. The optimal age to transition must be flexible	*It’s just a patient by patient basis, like if the patient feels comfortable moving on, they should move on, but if they don’t feel like they’re quite ready… so just ask the patient, I guess, and kind of gauge the patient’s feelings on it*. (Participant 11)
1b. There are varying levels of acceptable medical autonomy for survivors	*When I was in the pediatric clinic, my parents were super involved…And then I felt like the providers in that clinic were very open to that, and then I felt like maybe in the adult clinic it was kind of expected to be a little bit more just about the patient and less about the surrounding caregivers. I felt like they focused more on me as opposed to family*. (Participant 10)
2. Effective communication is critical to a successful transition	*I think them really communicating with me is what made it easiest. That’s the key to making a successful transition is having that communication, whether it be on the phone, through email, through physical mail, or any way that they can get that communication to you*. (Participant 12)
3. Survivors desire continuity during the transition	
3a. Reliable transfer of medical information to new providers is key	*One of the most important things would be having a seamless transition in the sense that you felt like the provider you were transitioning into was fully aware of your background and even some of the personal aspects of your care, and the whole package that you had in the pediatric clinic going into the adult clinic*. (Participant 9)
3b. Consistent provider(s) during the transition is helpful	*I think having a care team and a support system that really kind of goes on that journey with you…… [is] definitely important, just to have a transitional team that rides with you, even if you’re changing healthcare systems or if you’ve moved and just having a place to land wherever you go*. (Participant 14)
4. Comprehensive care means care that also addresses psycho-social well-being	
4a. Educational messages on health insurance are timely and very much needed	*I’m still 25, I work as a nurse and they offer me health insurance obviously. Because of all the health insurance issues and all the medications that I still need to take, I haven’t made the switch yet, so I’ll be doing that this fall, and I’m just worried about how the whole thing will go and what’s going to be covered and what’s not anymore.* (Participant 18)
4b. Support during life transitions is a necessary component of psycho-social support	*[The care team was] just really helpful in meeting those needs for me, whether it be social work, or legal, or things like that. Besides just the primary care piece, I think they just helped me with other issues that I was considering or thinking about as I was an adult by myself.* (Participant 7)

#### Theme 1: transition practices should be flexible and individually tailored

Multiple themes were identified around provider flexibility. This included flexibility on policies (e.g. age at transfer of care) as well as expectations for AYA developmental milestone attainment and even communication styles. Survivors repeatedly stated that there is no one “ideal” age to transfer to adult-centered care and emphasized the importance of waiting until the survivor is “ready” to transition care settings (Subtheme 1). In addition, our sample of survivors discussed the need for the care team to personalize their expectations of healthcare independence (Subtheme 2). There was a consensus that not all survivors are able to assume, at the same time, the same level of healthcare responsibility, both short-term and long-term.

#### Subtheme 1: the optimal age to transition must be flexible

From the survivor perspective, there was no standard age or set of milestones that determined when a patient was ready to transfer care to the adult care-setting. Some participants felt that in uncomplicated cases, the transition could be made at the patient and clinician’s discretion. According to one survivor “if it’s someone that doesn’t have any type of disabilities or anything, I would just think okay, you’re an adult now. We’re going to start transitioning you to adult [centered care].”(Participant 27) As maturation and agency vary from person to person, providers should be attentive to this variation and work together with survivors as a team to make sure that the survivor is ready:

“Everybody goes through something different in their life and they might not be ready to fully take responsibility for these check-ups. I think it’s just something that we should take seriously and make sure that we understand what it was that went on with us so we know what to expect.” (Participant 19)

As one survivor put it, providers should “communicate directly with … the patient or the family just to make sure they all are on the same page and they are willing to go ahead and try this transition, whether it’s when they’re 18 or whether it’s when they’re 21 ….” (Participant 26) Participants highlighted a personalized approach as most desirable: “It’s just a patient by patient basis, like if the patient feels comfortable moving on, they should move on, but if they don’t feel like they’re quite ready … so just ask the patient, I guess, and kind of gauge the patient’s feelings on it.” (Participant 11) The provider team being flexible was key to CCS: “If [the provider] can see that the patient may not be 100% ready by their body language or their communication … if they’re showing signs that they’re not really ready to transfer over, by observation. Maybe they can say maybe we’ll just wait a little bit before we transfer you over there. We can just keep you here and you’ll see your provider [here] until you feel a little bit more comfortable and ready to be transferred.” (Participant 23).

#### Subtheme 2: there are varying levels of acceptable medical autonomy for survivors

A desire for increased autonomy was voiced by several survivors when discussing successful transitions. Autonomy encompassed several key things including healthcare responsibility, independent decision-making, and self-efficacy. Survivors felt patients should be self-sufficient in reminding themselves about appointments and taking their medication without parental supervision and should have an awareness of their risks and necessary late effects surveillance. Most survivors concluded that a patient that is transitioning “should have a lot of responsibility for their healthcare” (Participant 3) and be comfortable with the added responsibility. This increase in autonomy was paralleled with decreased participation by parents. One survivor explained how their medical care changed during their own transition to adult care by saying, “Well, rather than just sitting [there] quietly, I ask a lot of questions. I can set up my own appointments [on] my own time, and I get called with the results rather than my parents. When I go and see other doctors…it’s my decision now; it’s not anyone else’s.” (Participant 11) Themes of autonomy and self-efficacy resonated with survivors as they felt that patients who are ready for the transition should be able to ask the right questions independent of their parents and have a good handle on their care needs.

Survivors also noted a change in the providers that promoted the development of healthcare autonomy: “When I was in the pediatric clinic, my parents were super involved … And then I felt like the providers in that clinic were very open to that, and then I felt like maybe in the adult clinic it was kind of expected to be a little bit more just about the patient and less about the surrounding caregivers. I felt like they focused more on me as opposed to family.” (Participant 10) Some survivors embraced the additional autonomy: “I felt like they were really looking at my needs as someone who was no longer a kid anymore, but someone who was in college, and adulthood, and had concerns about developing into a woman and gaining my independence. I felt like [the care team/clinic] were really looking at how they could help me with my survivorship as an adult.” (Participant 7) Patients were enthusiastic about engaging with their care team directly as a means to promote their medical autonomy. One patient said, “… as I got older, it was nice … for me to personally have the information, rather than having it all on my parents.” (Participant 8) However, this change in the patient-parent relationship to promote medical autonomy was revealed to be difficult by one survivor who explained, “[My parents] went to these appointments with me until I was 19, and my dad one day gave me an envelope with the date on it and he was like, ‘Here’s your appointment. I’ll go with you if you want; just let me know.’ and I didn’t [let him know]. I think they knew what was going on kind of, so they just called me later that day after my appointment to see how it all went. It took a very long time for them to be okay with it, and still, every time I have an appointment, they ask me if I want them to come and, if not, they just make sure that I call them afterwards and let them know how everything went. So they’re still very involved, they’re just not necessarily at the actual appointment.” (Participant 11) Another survivor shared, “I had relied on them [parents] to take care of me for a long time, and me taking the reins was a big step.” (Participant 1) Survivors did acknowledge that they wanted their parents to remain involved to some degree and preferred some level of parental involvement: “… one of the first things I did when I turned 18 was I signed that release form so that my parents could get any information because it’s good knowing that they can still check up on me. And if I have any problems and I really don’t know how to handle it, I can be like ‘Mom: can you call and deal with this for me?’” (Participant 21) In general, finding a balance between parent involvement and independence is an essential component to a successful transition but must also be achieved through an individualized approach to promote survivor medical autonomy.

#### Theme 2: effective communication of medical information is critical to a successful transition

Survivors expressed that they wanted information from providers that explained why they needed to transition and what to expect during their adult-centered visits: “I guess being informed of what is expected of me and being informed on what the future holds in terms of visits, what type of bloodwork I need, what type of scans, just regular maintenance things. I really enjoy being informed, and I like to know what to expect.” (Participant 28) One survivor commented that “the most important [thing is] … just letting you know how things are going to go.” (Participant 12) The survivor continued to say that providers should understand that everything was so routine during pediatric-centered care so as a result, preparing survivors for having more responsibility over appointments helps survivors know what to expect. Clear communication that utilizes multiple modalities seemed most critical, as one survivor put it: “I think them really communicating with me is what made it easiest. That’s the key to making a successful transition is having that communication, whether it be on the phone, through email, through physical mail, or any way that they can get that communication to you.” (Participant 12) An integral component of effective communication was how accessible providers were to patients, “I had names of people, which was also helpful. It wasn’t just a department; there was the name of a person I could go to.” (Participant 7).

At times, even over-communication of information from providers to survivors is appropriate:

“I don’t think there’s ever a point where I’m given too much information and all the information they have would be great. I guess I haven’t really been sat down and told all the things I could’ve had issues with. Actually, I guess I was looking at my notes, or something and I saw all of the medications, or the chemotherapy drugs I was on and all the problems that could occur, and things that I have to look for down the road, and I wasn’t aware of most of them. I’m sure they told me at some point, but it hadn’t sunk in, so yeah, I guess err on the side of throwing more information at me than I could possible need.” (Participant 1)

This desire for large volume information is well suited to the longitudinal nature of survivor-focused care where discussions can occur over several years by several team members.

In addition to conversations regarding late effects risk, surveillance and health promotion, some survivors said that they would have liked to dedicate at least one appointment to a conversation with their provider about what to expect during the transition so that they had more information about the transition before the time of transfer. Preparing survivors for the transition was repeatedly highlighted: “I would think just to let people know to be aware that they are accountable for it. My mom had to tell me. She was like oh yeah, they’re not going to do that again, because you’re an adult now so it’s all on you. It’s like okay, cool. I’m like if she hadn’t told me, I don’t know how I would’ve known. Just like for maybe the first appointment to kind of let you know.” (Participant 12) Survivors wanted to work with their providers to design a clear and feasible plan for the transition. One survivor suggested written materials be given to survivors prior to and during the transition detailing the process and upcoming changes. As one young adult put it, “I love going to the library and reading, and maybe they could have given me some information packets about like what was going to happen, what would be done, what the transitioning looked like …” (Participant 15) Effective communication was often reported by survivors to be a critical element in their own successful transition: “Yes, both [transitional care teams] were really helpful. They just communicated really well with me like okay, we’re going to be doing this now. We’re going to move you from here to here. Here are the people you need to contact if you have any questions. You can call at any time or email if you need more resources, or here’s some other things you can do to make the transition easier.” (Participant 21).

#### Theme 3: survivors desire continuity during the transition

The young adult CCS repeatedly expressed that they expected continuity in their medical care. From this, two subthemes emerged: the first subtheme was that CCS want their new adult-centered care team to have an in-depth knowledge of their medical history and other relevant survivor-related information. The second subtheme was that the survivors wanted to establish a consistent and long-term relationship with their new provider, just like they had with their pediatric-centered survivorship care team. Patient’s wanted to feel like their providers cared about their health. This was exemplified by one patient’s narrative about what made their experience so positive during transitional care: “Yeah, seeing past people, and even the people who take blood samples, just seeing them, I loved doing that. I’ve been down there for fifteen-plus years, and I love doing that because it makes you feel like you’re part of the community. People know who you are and that you’re important.” (Participant 2) This survivor’s experience being part of a community directly impacted patient satisfaction with the transition to adult-centered survivor-focused care.

#### Subtheme 1: reliable transfer of medical information to new providers is key

For survivors, one of the most important aspects of a successful transition was the transfer of medical information to new providers. This included survivors feeling confident that every new provider would consistently receive the information necessary to be informed of their past medical history. One young adult described this by saying, “One of the most important things would be having a seamless transition in the sense that you felt like the provider you were transitioning into was fully aware of your background and even some of the personal aspects of your care, and the whole package that you had in the pediatric clinic going into the adult clinic.” (Participant 9) Another survivor elaborated on this by articulating that a survivor should feel like “a doctor or facility is picking up where you left off. They know your history, what you’re dealing with, [and] what’s next in terms of follow-up.” (Participant 28) Participants also detailed a level of depth that was critical to the consistent transfer of medical history during the transition, including psychosocial well-being. The seamless and consistent transfer of these details had survivors reporting that it boosted their confidence that the appropriate care would be provided:

“I really like how the providers in the adult clinic seemed to know everything about me before I even got there. I know they get the electronic records and stuff, but even on a deeper level than that, I felt like they were already very aware of me. I felt like some conversation had taken place, maybe, between the peds and the adult providers so that the adult providers knew exactly what I was doing, and school, and knowing how my experiences affected what I wanted to do. They understood some of the aspects of my experience and how that might impact my attitude towards other follow-up and stuff like that. I felt they did a pretty good job with continuity.” (Participant 9)

“I’ve been used to going to doctors all the time, so I guess the biggest concern I have is kind of the transfer of knowledge from one doctor to another. That’s one thing I run into as an adult. When I go to the doctor, it’s like oh yeah, I’ve had all of these issues, so not having to re-explain things is what I would consider a successful transition.” (Participant 1)

#### Subtheme 2: consistent provider(s) during the transition is helpful

Several participants verbalized concerns that a change in provider was a set-up for inconsistent transfer of survivor-focused medical history from the pediatric to adult-setting. “It would be nice to continue seeing the same oncologist, just, again, because they’re more familiar with who I am and my diagnosis and my follow-up, and everything in-between …” (Participant 28) Another survivor provided additional insight, saying: “I think having a care team and a support system that really kind of goes on that journey with you … … [is] definitely important, just to have a transitional team that rides with you, even if you’re changing healthcare systems or if you’ve moved and just having a place to land wherever you go.” (Participant 14) Survivors did acknowledge limitations: “I think I’ve only seen my long-term follow-up person twice or maybe three times, but I think I saw two different people. Yeah, actually if I could continue seeing the same person that would probably be better, but the scheduling is such a problem that I’ll go see whoever. I would prefer to see the same person every time.” (Participant 1) Despite this reality, respondents reiterated in several interviews that they “saw a lot of doctors in my time there, and it was really nice to have [someone] there for a period of time so that I had a relationship with someone as opposed to coming again and seeing someone different all the time and so I do think that would be good to just have some sort of person to transition me.” (Participant 7).

#### Theme 4: comprehensive care means care that also addresses psycho-social well-being

Survivors described comprehensive survivorship care as including support for psychosocial and general well-being. These survivors were receiving care in a model that provided regular and longitudinal care from a pediatric social worker experienced in the issues faced by childhood cancer survivors.

#### Subtheme 1: educational messages on health insurance are timely and very much needed

For many participants, fully understanding their health insurance coverage was a stressor, particularly how it related to an emerging adults’ interaction with insurance issues. One survivor said, “I’m still 25, I work as a nurse and they offer me health insurance obviously. Because of all the health insurance issues and all the medications that I still need to take, I haven’t made the switch yet, so I’ll be doing that this fall, and I’m just worried about how the whole thing will go and what’s going to be covered and what’s not anymore.” (Participant 18) This insecurity was voiced by another survivor who said, “I was going through a time where I would be leaving my parents’ insurance, and so I was concerned about getting health insurance and if I would even qualify for health insurance as someone who [had] cancer.” (Participant 7) But the survivor continued to say “[The care team was] just really helpful in meeting those needs for me, whether it be social work, or legal, or things like that. Besides just the primary care piece, I think they just helped me with other issues that I was considering or thinking about as I was an adult by myself.” (Participant 7) Other survivors also commented on how confusing health insurance plans can be: “I had to have all my labs and tests done elsewhere, and then sent over to [the childhood cancer survivor program]. I think that’s the biggest thing, I guess, is not having to worry about the backside of things and more so worry about what’s going on and the actual results of all the testing.” (Participant 5) Navigating insurance issues with referrals also came up as a challenge for survivors: “The more specialized visits; I know I will need to get a referral from my provider that’s listed on my insurance to get an orthopedic visit scheduled, so it’s a lot of back and forth, mainly because of the insurance issues and what they accept and what they don’t accept. But I think everyone I see at the university are doing what they can [to help].” (Participant 28).

#### Subtheme 2: support during life transitions is a necessary component of psycho-social support

Life transitions that occur around the time of care transitions often lend themselves to support from psycho-social providers such as social workers. This includes the anxieties generally associated with young-adults achieving developmental milestones but also the amplified effect on overall well-being for CCS during these potentially stressful times. Survivors highlighted the importance of support, including peer-support, when it came to psychosocial well-being. One survivor explained: “I know another thing that really helped is to have the survivor conference, the annual conference. That’s something that I’ve gone to for the last few years, and I’ve brought my family too. So, just feeling that all these other people that are receiving care through the same place for all different kinds of situations. So that was very helpful because it feels more like a community than an individual case, so I think that’s something that’s really helpful and I really look forward to going and experiencing that every year.” (Participant 14) This extended to parents as well: “a lot of times a disease will affect the mom and dad in ways more than the child, or the teenager, or the adult with it, and I think it’s a good idea for the family dynamic to have the caregiver, the parent, the father, the mother, to have that level of support as well.” (Participant 3) Financial toxicity was addressed by survivors most often in the context of another life transition: transitioning to college and the associated costs. Several participants commented on the psycho-social support that they had received informing them of college scholarships available for childhood cancer survivors: “I know there are scholarships for people, for survivors, and that would be nice to have some of that information for grad school. I don’t know, because I see a social worker every time. I don’t know if that’s the thing in the scope of something that they would do, but … that would be cool.” (Participant 1).

## Discussion

For all children, the transition to young adulthood is fraught with challenges and uncertainties. The medical consequences of these issues become amplified in a population of CCS, just as they do for many patients with chronic diseases of childhood. Through the presented data, the collective voice of CCS expresses a clear and granular desire that continuity be prioritized through all levels of their care around and during the transition from pediatric- to adult-settings but that it also be balanced with the appropriate flexibility. For the CCS studied here, a successful transition includes effective communication thorough the collection, dissemination and integration of not just their past medical history but also their past psycho-social history. Communication must therefore be both consistent and flexible which is where specialized personnel can play a critical role in realizing these priorities for CCS in an effort to ensure a smooth transition of care.

Our findings have been echoed by the previous literature in this area. Cohorts of childhood cancer survivors from all over the world have reported the importance of communicating educational and informational messages to survivors both effectively and in multiple formats [26–31]. Early introduction of the transition of care as well as strong provider collaboration are also often cited as being critical [26–31]. Our work adds to this body of literature by applying these shared care preferences to a novel and more specific aspect of survivor care, their psychosocial well-being.

The challenge of delivering consistent yet flexible comprehensive care is magnified due to the diversity of how this care is delivered to CCS both across and within institutions and their respective healthcare systems. A consistent transfer of medical information was desired as well as a consistent member of the care team being present across both the pediatric and adult care-settings. The complexity in building both continuity and flexibility in transition care models may require the utilization of personnel within healthcare systems that possess expertise in the coordination of complex care. Whether this role is filled by providers with nursing or social work expertise is perhaps less relevant and may vary depending on the healthcare system. More importantly, the person in this role must be intimately familiar with both the pediatric and adult institutions and how to function within their varying models of care and varying locations within their healthcare system. The Academy of Oncology Nursing and Patient Navigators call this role a patient navigator (PN) and defines it as the critical person on the multidisciplinary team that serves as a primary point of contact for the patient and works with other members of the care team to coordinate care for the patient and provides important perspective on logistical, structural and social needs of the patient as well as cultural considerations, patient values and care preferences [32]. The PN provides support with all aspects of cancer care including financial challenges and other psychosocial and community issues. Integrating this role into the routine care of CCS would align with the findings of our work and lend itself to optimizing the transition from pediatric- to adult-centered care across a healthcare system. Despite a lack of evidence on the direct impact of the PN role in the care of CCS, there are metrics for navigation practices that have specific impacts on the quality of patient care that is delivered [33]. A related future area of research would entail both clinical outcomes and quality improvement efforts aligning with a truly multi-disciplinary health services approach necessary to illustrate the benefits in the transitional care of AYA-aged CCS.

One particular challenge in providing flexible transitional care for CCS is the delicate and evolving balance of involving parents to the appropriate degree. While the provider team aims to empower AYAs with healthcare responsibility, there is also a simultaneous distancing from parents. But clinical teams should be aware that survivors voiced a clear comfort in knowing that their parents provided a safety net of sorts for both communication and medical decision making. At the same time, if parents are too involved then they may be a detriment to growing a survivor’s healthcare independence. There likely is a balance of how and when to involve parents in AYA CCS care that can be best understood by providers that have long-standing relationships with both the survivor and the parent. This may be another area where personnel within the healthcare care system with patient navigation expertise could also effectively navigate the inter-personal dynamics of each survivor and their parents to optimize survivor growth in self-efficacy and attainment of appropriate healthcare responsibility.

An initial limitation to the findings of this study is the selection bias present through having a participant sample that included one medical center and thus one model of transitional care. CCS provided authentic details of their lived-experience but their survivor-focused care was reflective of one particular model of care that included emphasis on provider familiarity through continuity. The model of care had consistent providers throughout the transition from pediatric- to adult-centered survivor care and this may have led to a participant bias where survivors expressed a desire for continuity because that was a key principle of the transitional care that they received. In addition, their voices did not represent the survivors lost to follow-up and not actively receiving survivor-focused care. In awareness of this participant bias, survivors from all time points in the transitional period were included in this study. Some survivors were not yet transferred to adult-centered care, some had just recently been transferred and others had completed their transition firmly placing them in the adult care-setting.

The message from CCS, as illustrated in this work, is loud and clear. To effectively and efficiently keep CCS in the healthcare system as they age through adolescence and into young adulthood, survivors want providers that are flexible in multiple ways yet consistent with their communication and also able to facilitate comprehensive care that is pro-active instead of reactive. Models of care must be responsive to the ever-changing needs of CCS and possess the diversity of expertise to address both the physical and psycho-social concerns. The patient navigator role may be the ideal personnel within the healthcare system to operationalize these patient-centered values. In order to deliver such care, novel research that focuses on the learning health system (LHS) will be necessary [34]. This approach uses a value-based care framework where “internal data and experience are systematically integrated with external evidence, and that knowledge is put into practice. As a result, patients get higher quality, safer, more efficient care, and healthcare delivery organizations become better places to work.” Such research would be critical to implement and evaluate the role of a patient navigator through real-time rapid-cycle improvement efforts and other related quality improvement work. And for CCS, LHS research would translate to more meaningful longitudinal survivor-focused care that can then result in life-long early detection or prevention of long-term complications from childhood cancer therapies.

## Conclusions

Models of care for CCS should be built to provide, or seamlessly facilitate, continuous survivor-focused care across the age continuum. A longitudinal relationship with a survivor-focused provider can help promote the values that CCS report as important in transitioning care from pediatric- to adult-centered care.

## Supplementary information

**Additional file 1.** Interview Guide.pdf; Title of Data: Online Resource 1; Description of Data: Questionnaire.

## Data Availability

The datasets generated and/or analysed during the current study are not publicly available due to IRB restrictions but are available from the corresponding author on reasonable request.

## Abbreviations

AYA: Adolescent and young adult
CCS: Childhood cancer survivors
LTFU: Long-term follow-up
CCSP: Childhood Cancer Survivor Program
IRB: Institutional review board
PN: Patient navigator
LHS: Learning health system
