# Supplementary material for: Identifying metrics of success for transitional care practices in childhood cancer survivorship: a qualitative interview study of survivors
Source: BMC Cancer. 2020 Sep 21;20:898. doi: 10.1186/s12885-020-07360-9 (PMC7507711; doi:10.1186/s12885-020-07360-9)
Supplement: Supplementary file 1 — Additional file 1. Interview Guide.pdf; Title of Data: Online Resource 1; Description of Data: Questionnaire. [file 12885_2020_7360_MOESM1_ESM.pdf]

Interview Guide-Survivor

Hello, my name is [______], and I am calling from the University of Minnesota regarding the study on successful transitions in survivorship care that you discussed with our childhood Cancer Survivor Program. Is this still a good time to run through the interview?

IF NOT-Reschedule.

IF SO- Great! Thank you for your interest in this study. There are a few things I'd like to remind you about before we start.

1. *First, we expect this interview to take about 15-30 minutes to complete, depending on what you have to say. Therefore, please find a quiet and comfortable spot to do the interview. This interview is being recorded.*
2. *Everything we talk about will only be used for research purposes. All interviews will be anonymous and compiled together so no one will know which interview was yours. And the interviews will not be shared with anyone outside the study team. Your participation is also completely voluntary.*

As you probably heard from Karim Sadak, the principal investigator of this study and Director of our childhood Cancer Survivor Program, we are interested in learning about your perspective on successful transitions of survivorship care from our child-focused clinic to our adult clinic. This includes your very important perspective as the survivor..

**** When we talk about transition of survivorship care, I'm referring to the change from receiving your survivorship care in the Journey Clinic at the Children's Hospital on the West Bank/Riverside Campus to attending the adult clinic at the Masonic Cancer Center on the East Bank. ****

So the interview will cover a few things: First, we will ask about what you think would help or would have helped in making your transition from Journey clinic to the adult clinic most successful. Then we will briefly ask you to describe what you believe your parent or caregiver would see as a successful transition and lastly we'll talk about the same thing with you describing the opinion of your survivorship doctor or nurse practitioner.

We will tape record this conversation so we can be sure we get everything you say just the way you said it. We are really excited that you have agreed to participate because we think we can learn a lot about successful transitions from people like you that are living it and going through this first hand. So in this conversation, speak freely and from your experience and I'll get us through this as quickly as possible. Does that sound ok to you?

Do you have any questions before we start? Do you have any questions about your participation in the study?

1.	First, I want to start by asking some general questions:<br>How old are you and what cancer(s) did you have?	
2.	So thinking about your experience receiving survivorship care and growing up in general, how would you describe what a survivor would see as a successful transition?	
2a.	ELICIT UNDERSTANDING:<br>• Can you tell me more about that?<br>• Can you tell me what you mean when you say you [that]?	
2b.	PROBES<br>• What do you think are some of the most important aspects leading up to the transition? These could be things that you have to accomplish before or after the transition or even during it.<br>• I notice you haven't said anything about<br>o wanting an age-appropriate office setting for you<br>o feeling ready to transition<br>o achieving a particular level of understanding of your current late-effects and any potential future risks<br>o your insurance being accepted at the new adult program<br>o wanting to see someone who knows about their childhood cancer history<br>o wanting to see multiple specialists all at once during one visit<br>o wanting to see a familiar face after the transition	
3.	How do you think your parent/caregiver would describe a successful transition?	
3a.	ELICIT UNDERSTANDING:<br>• Can you tell me more about that?<br>• Can you tell me what you mean when you say you [that]?	
3b.	PROBES:<br>• I notice you haven't said anything about<br>o them wanting you achieving a certain level of understanding re your health risks<br>o your parents themselves feeling ready for the transition<br>o your parents wanting you to get help with career planning<br>o your parents wanting you to get help with health insurance issues<br>o your parents wanting you to schedule your own appointments	
4.	Lastly, how would you describe what your doctor or nurse practitioner sees as a successful transition?	
4a.	ELICIT UNDERSTANDING:<br>• Can you tell me more about that?<br>• Can you tell me what you mean when you say you [that]?	
4b	PROBES:<br>• I notice you haven't said anything about<br>o them assessing your readiness for the transition<br>o them documenting some level of your education regarding your treatment history<br>o them updating your contact information to inform you of future research opportunities<br>o financial issues like insurance coverage or how that changes as you get older<br>o them involving your primary care provider<br>o a specific age when you think you should be transitioned	
5.	Is there more? Anything else you feel we missed?	

Finish by thanking them for their time and participation. Ask if they would be willing to have us call them back if we need to clarify information. Also leave them with an invitation to contact Dr. Sadak (612-626-2778, ktsadak@umn.edu) with any questions or comments regarding the interview, the project or anything related to this topic.
